# C-terminal–modified LY2510924: a versatile scaffold for targeting C-X-C chemokine receptor type 4

**DOI:** 10.1038/s41598-019-51754-0

**Published:** 2019-10-25

**Authors:** Kentaro Suzuki, Takashi Ui, Akio Nagano, Akihiro Hino, Yasushi Arano

**Affiliations:** 1 RI Research Department, Research Division, FUJIFILM Toyama Chemical Co., Ltd., 453-1, Shimo-Okura, Matsuo-Machi, Sammu-City, Chiba 289-1592 Japan; 2 Department of Molecular Imaging and Radiotherapy, Graduate School of Pharmaceutical Sciences, Chiba University, 1-8-1, Inohana, Chuo-ku, Chiba 260-8675 Japan; 3 Research Department, FUJIFILM RI Pharma Co., Ltd., 453-1, Shimo-Okura, Matsuo-Machi, Sammu-City, Chiba 289-1592 Japan

**Keywords:** Positron-emission tomography, Cancer imaging, Radiotherapy, Acute lymphocytic leukaemia, Diagnostics

## Abstract

C-X-C chemokine receptor type 4 (CXCR4) constitutes a promising target for tumor diagnosis and therapy. Herein, we evaluate a new 1,4,7,10-tetraazacyclododecane-1,4,7,10-tetraacetic acid (DOTA)-conjugated CXCR4 antagonist derived from LY2510924, FRM001, and its metal complexes as CXCR4-targeting probes. FRM001 was synthesized by modifying the C-terminus of LY2510924 with maleimido-mono-amide-DOTA via a cysteine linker. FRM001 exhibited CXCR4-specific binding with an affinity similar to that of the parental LY2510924. The binding affinity of FRM001 remained unchanged after complexation with Ga, Lu, and Y. The internalization of ^67^Ga-FRM001 into the cells was hardly observed. In mice biodistribution studies, ^67^Ga-FRM001 exhibited high accumulation in the tumor and the liver with rapid elimination rates from the blood. The hepatic accumulation of ^67^Ga-FRM001 was preferentially and significantly reduced by co-injecting a CXCR4 antagonist, AMD3100. The C-terminal–modified LY2510924 would constitute a versatile scaffold to develop CXCR4-targeting probes or therapeutics for tumor imaging or therapy.

## Introduction

C-X-C chemokine receptor type 4 (CXCR4) is a highly conserved seven-span transmembrane G protein–coupled receptor that binds the ligand stromal cell-derived factor 1 (SDF-1; also called CXCL12)^1^. As CXCR4 is overexpressed in more than 23 types of human cancers and contributes to tumor growth, angiogenesis, metastasis, and therapeutic resistance^2^, CXCR4 represents a promising target for tumor diagnosis and therapy^3,4^. Indeed, CXCR4 antagonists have been developed to target the CXCR4–CXCL12 axis^5^. CXCR4 antagonists have also been evaluated as vectors to deliver radiation or cytotoxic drugs to tumors for imaging and/or therapy^6–8^.

A variety of radiolabeled CXCR4-targeting agents have been developed such as gallium-68 (^68^Ga)-NOTA-NFB and copper-64 (^64^Cu)-AMD3100 (plerixafor)^9,10^. Among them, FC131 (*cyclo*[_D_-Tyr-Arg-Arg-2-Nal-Gly]) derivative ^68^Ga-pentixafor (*cyclo*(_D_-Tyr-_D_-[*N*Me]Orn-[AMBS-DOTA]-Arg-2-Nal-Gly) [where AMBS represents 4-(aminomethyl) benzoic acid)] exhibits excellent CXCR4-targeting properties and low hepatic accumulation^11,12^. Positron emission tomography (PET) imaging with ^68^Ga-pentixafor visualizes CXCR4 expression in multiple myeloma^13–17^, leukemia^18,19^, adrenocortical carcinoma^20^, glioblastoma^21^, small-cell lung cancer^22,23^, non-small-cell lung cancer^24^, lymphoproliferative diseases^25^, neuroendocrine tumors^26,27^, extranodal marginal zone lymphoma^28^, and esophageal adenocarcinoma^29^. However, the application of pentixafor to β^–^-emitting routinely used radionuclides such as lutetium-177 (^177^Lu) and yttrium-90 (^90^Y) for cancer endoradiotherapy is inappropriate due to the reduction in CXCR4-binding affinities^30^. As an alternative to pentixafor, pentixather has been developed for labeling with β^−^-emitting radionuclides, where _D_-Tyr in pentixafor is replaced with iodo-_D_-Tyr. Endoradiotherapy with ^177^Lu/^90^Y-pentixather is well tolerated and exerts antitumor activity against advanced-stage multiple myeloma^13,14^ and acute leukemia^19^. These studies have indicated that radiolabeled CXCR4-targeting agents are potentially useful for the diagnosis and therapy of tumors when combined with radionuclides of clinical relevance. However, the radiolabeled pentixather derivative also shows high hepatic accumulation^31^.

**Figure 1:**
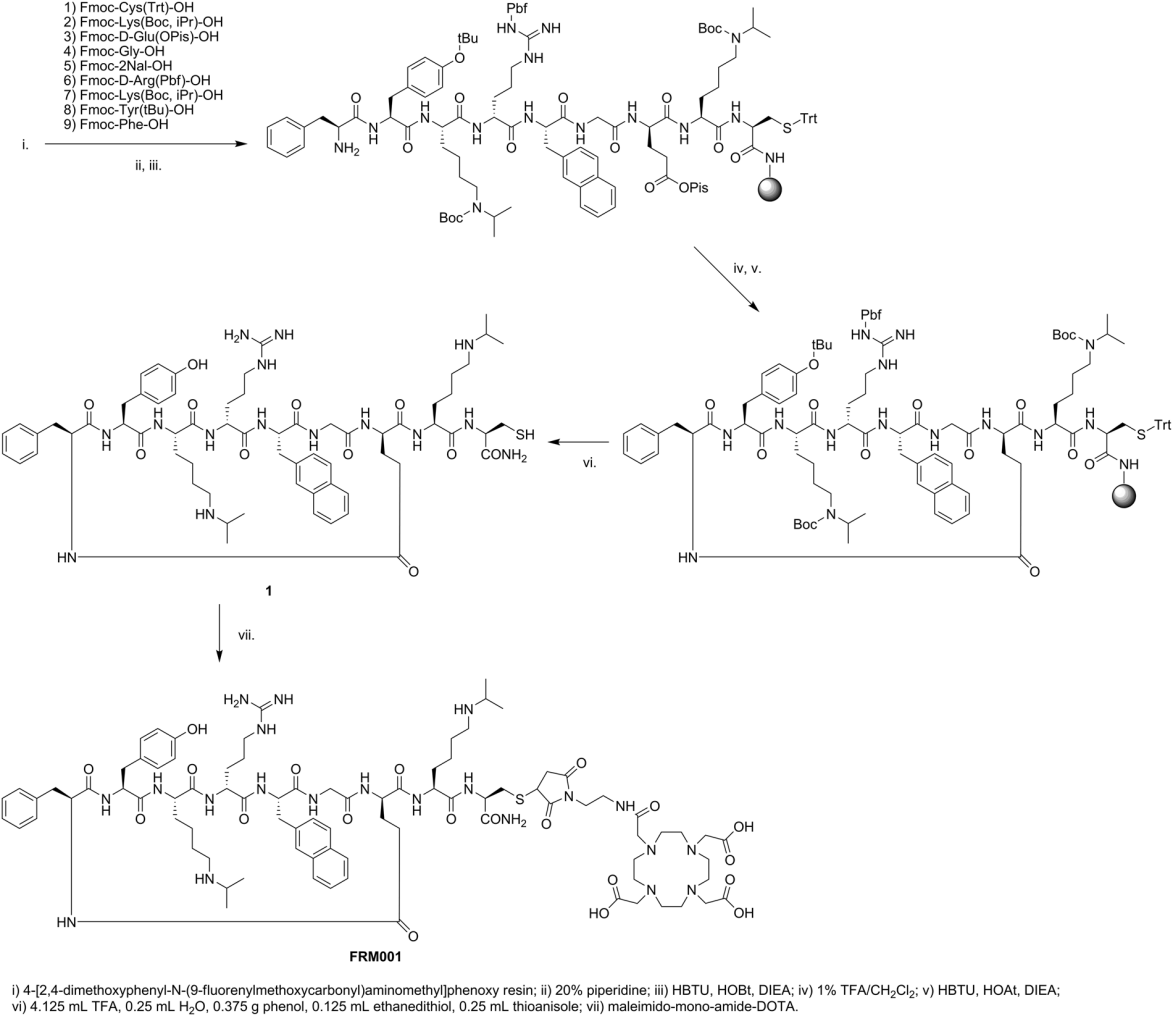
Synthetic procedure for FRM001.

In this study, we searched for an alternative CXCR4-targeting scaffold that possesses high CXCR4 binding affinity and preserves its binding affinity after conjugation of a chelating molecule and subsequent complexation reaction with metallic radionuclides such as ^67/68^Ga, ^90^Y, and ^177^Lu. We selected LY2510924 (*cyclo*[Phe-Tyr-Lys(iPr)-_D_-Arg-2-Nal-Gly-_D_-Glu]-Lys(iPr)-NH_2_) as a CXCR4-binding motif of choice considering its high CXCR4-binding affinity and *in vivo* stability^32^. LY2510924 was conjugated with 1,4,7,10-tetraazacyclododecane-1,4,7,10-tetraacetic acid (DOTA) via a cysteine linker to produce FRM001 for radiolabeling with ^68^Ga, ^177^Lu, ^90^Y, and the actinium-225 (^225^Ac)^33^, as shown in Fig. 1. Consequently, we compared the CXCR4-binding affinity of FRM001 and its natural gallium (Ga), natural lutetium (Lu), and natural yttrium (Y) complexes in CCRF-CEM, a human acute lymphoblastic leukemia cell line expressing endogenous CXCR4^34^, using LY2510924 and other CXCR4-targeting molecules as references. The ability of FRM001 to target CXCR4 was also evaluated with ^67/68^Ga-FRM001 in a mouse model.

## Materials and Methods

### Materials

The following CXCR4 antagonists were purchased from commercial sources, as follows: AMD3100 and AMD3465 hexahydrobromide from AdooQ BioScience (Irvine, CA); LY2510924 and BKT140 from MedChemExpress (Monmouth Junction, NJ); and FC131 from FUJIFILM Wako Pure Chemical (Osaka, Japan). Additionally, maleimido-mono-amide-DOTA was obtained from Macrocyclics (Plano, TX), while GaCl_3_, LuCl_3_, and YCl_3_ were purchased from Mitsuwa Chemicals (Osaka), Strem Chemicals (Newburyport, MA), and FUJIFILM Wako Pure Chemical, respectively. ^67^GaCl_3_ was obtained from FUJIFILM Toyama Chemical (Tokyo, Japan), while a ^68^Ge/^68^Ga generator was purchased from ITG Isotope Technologies Garching (Munich, Germany). Iodine-125 (^125^I)-SDF-1α was purchased from PerkinElmer (Waltham, MA). Other reagents and solvents were purchased from major suppliers and were used without further purification unless indicated.

The radio-TLC analysis was performed on TLC silica gel 60 RP-18 F_254_s (Merck, Darmstadt, Germany) developed with a 1:1 (v/v) mixture of 2 M of ammonium acetate solution and acetone. The strips were analyzed by a GITA Star gamma-TLC scanner (Elysia-Raytest, Straubenhardt, Germany). Radio-HPLC was performed by using the Alliance 2695 HPLC system (Waters, Milford, MA), connected with a 2489 ultraviolet/visible light detector (Waters) and a GABI Star gamma radio detector (Elysia-Raytest). A TSKgel ODS-80Ts QA (5 μm, 4.6 × 250 mm) column (Tosoh, Tokyo) with a flow rate of 1 mL/min was used. Acetonitrile [0.1% trifluoroacetic acid (TFA)] in water (0.1%TFA) was used as the mobile phase with the following multistep gradient: 0–20 min, 20–40% acetonitrile (0.1%TFA); 20–21 min, 40%–100% acetonitrile (0.1%TFA); and 21–30 min, 100% acetonitrile (0.1%TFA).

Matrix-assisted laser desorption/ionization time-of-flight mass spectrometry (MALDI TOF-MS) was performed on a Microflex system (Bruker, Billerica, MA), while electrospray ionization-mass spectrometry (ESI-MS) was performed using an LCMS-2010EV system (Shimadzu, Kyoto, Japan). Radioactivity was measured using a 2480 WIZERD^2^ automatic gamma counter (PerkinElmer). PET images were acquired using an Inveon PET scanner (Siemens, Munich).

### FRM001 synthesis

The synthesis of FRM001 (*cyclo*[Phe-Tyr-Lys(iPr)-_D_-Arg-2-Nal-Gly-_D_-Glu]-Lys(iPr)-Cys(maleimido-mono-amide-DOTA)-NH_2,_ molecular weight: 1,819.13) was entrusted to Scrum (Tokyo) (Fig. 1). *cyclo*[Phe-Tyr-Lys(iPr)-_D_-Arg-2-Nal-Gly-_D_-Glu]-Lys(iPr)-Cys-NH_2_ (1) was synthesized by solid-phase-peptide synthesis using Fmoc-protected amino acids. Side-chain protecting groups were Fmoc-Cys(Trt)-OH, Fmoc-Lys(Boc, iPr)-OH, Fmoc-D-Glu(OPis)-OH, Fmoc-Gly-OH, Fmoc-2Nal-OH, Fmoc-D-Arg(Pbf)-OH, Fmoc-Lys(Boc, iPr)-OH, Fmoc-Tyr(tBu)-OH, and Fmoc-Phe-OH. Peptides were extended using 4-[2,4-dimethoxyphenyl-N-(9-fluorenylmethoxycarbonyl)aminomethyl]phenoxy resin as a starting material. After constructing the peptide, the cyclization of the peptide was conducted on the resin by removing the OPis-protecting group with 1% TFA/CH_2_Cl_2_ and subsequent conjugation with the amine group of N-terminal phenylalanine in the presence of HBTU/HOAt/DIEA. The resulting peptide was treated with a TFA cocktail solution (4.125 mL of TFA, 0.25 mL of H_2_O, 0.375 g of phenol, 0.125 mL of ethanedithiol, and 0.25 mL of thioanisole) to isolate the crude peptide. After purification by reversed-phase (RP)-HPLC, the resulting peptide was conjugated with a maleimido-mono-amide-DOTA to prepare FRM001. The peptide was purified by RP-HPLC and lyophilized. MALDI TOF-MS: calculated for (C_87_H_127_N_21_O_20_S): 1,817.93; found: *m/z* = 1,818.435 [M + H]^+^.

### Ga-FRM001 synthesis

FRM001 and GaCl_3_ were dissolved in 0.5 M of sodium acetate buffer (pH: 5) to prepare a solution containing 2.7 mM of FRM001 and 54 mM of GaCl_3_. After 30 min incubation at 45 °C, Ga-FRM001 was purified with RP-HPLC and then lyophilized. ESI-MS: calculated for (C_87_H_125_GaN_21_O_20_S): 1,884.84; found: *m/z* = 943 [M + 2H]^2+^.

### Lu-FRM001 synthesis

Lu-FRM001 was synthesized according to the procedure employed for Ga-FRM001. Briefly, a solution of 2.7 mM of FRM001 and 54 mM of LuCl_3_ was incubated for 30 min at 45 °C. Lu-FRM001 was obtained after purification by RP-HPLC and subsequent lyophilization. ESI-MS: calculated for (C_87_H_124_LuN_21_O_20_S): 1,989.85; found: *m/z* = 996 [M + 2H]^2+^.

### Y-FRM001 synthesis

FRM001 and YCl_3_ were also prepared according to the procedure as mentioned above. Y-FRM001 was purified with RP-HPLC and then lyophilized. ESI-MS: calculated for (C_87_H_124_N_21_O_20_SY): 1,903.81; found: *m/z* = 953 [M + 2H]^2+^.

### Radiolabeling of FRM001 with ^67^Ga

A solution of ^67^GaCl_3_ in 0.3–0.6 M of HCl was added to a solution of FRM001 in 0.5 M of sodium acetate buffer (pH: 5). The mixed solution containing 0.3 GBq/mL of ^67^Ga and 0.1 mM of FRM001 with a pH of 5 was incubated for 10 min at 95 °C. ^67^Ga-FRM001 was absorbed on a Sep-Pak C18 Vac cartridge (Waters), washed with water, and eluted with acetonitrile containing 0.1%TFA. After removing the organic solvent, ^67^Ga-FRM001 was reconstituted in 50% ethanol. Radiochemical purity was determined with radio-TLC and radio-HPLC.

### Radiolabeling of FRM001 with ^68^Ga

An eluate from a ^68^Ge/^68^Ga generator was added to a solution of FRM001 dissolved in 1 M of sodium acetate buffer (pH: 5). The resulting solution contained 0.2–0.3 GBq/mL of ^68^Ga and 0.01 mM of FRM001 at a pH of 5. The mixture was incubated for 10 min at 95 °C, and radiochemical purity was determined by radio-TLC and radio-HPLC.

### Lipophilicity

A solution of ^67^Ga-FRM001 (168 kBq, 56.8 pmol of FRM001) in 0.5 mL of 1-octanol was added to a 0.5 mL solution of phosphate-buffered saline (PBS) (pH 7.4). The mixture was vortexed and rotated at room temperature for 5 min. After centrifugation at 13,000 × g for 5 min, the radioactivity concentrations in samples of both the aqueous and organic phases were determined by using a gamma counter, and the logP_ow_ values were calculated.

### Cell culture

A human acute lymphoblastic leukemia cell line, CCRF-CEM^35^, was obtained from the JCRB Cell Bank (JCRB0033; Osaka) and maintained in Roswell Park Memorial Institute 1640 (RPMI-1640; Thermo Fisher Scientific, Waltham) supplemented with 10% (v/v) fetal bovine serum, 100 units/mL of penicillin, and 100 μg/mL of streptomycin. The cells were cultured in a humidified atmosphere of 95% air and 5% carbon dioxide at 37 °C. Passages were performed twice a week.

### Competitive binding assay

CCRF-CEM cells and ^125^I-SDF-1α were used for the competitive binding assay. The competitive binding assay was performed in a 96-well MultiScreen HTS FB plate (Merck) treated with 0.1% polyethyleneimine. RPMI-1640 containing 1% bovine serum albumin (BSA) and 0.05% Tween20 was used for the assay buffer. Briefly, 200-μL reaction mixtures containing CCRF-CEM (0.5 × 10^6^ cells), ^125^I-SDF-1α (1 kBq), SDF-1α (0.05 pmol; R&D Systems, Minneapolis, MN), and varying concentrations of CXCR4 antagonists were incubated at room temperature for one hour. After the incubation, the cells were washed three times with the assay buffer, and radioactivity counts of cells were determined with a gamma counter. The IC_50_ values were calculated by using GraphPad Prism version 5.04 (GraphPad Software, San Diego, CA).

### Stability assessment

A ^67^Ga-FRM001 solution was diluted 11-fold in human serum (Access Biologicals, Vista, CA), and the solutions were then incubated at 37 °C. After zero, 1.5, and four hours, 0.05-mL aliquots were precipitated and centrifuged at 13,000 × g for 5 min after adding 0.15 mL of acetonitrile (0.1%TFA). The supernatants were then analyzed using radio-HPLC.

### Binding assay

The binding assay was performed by using a procedure similar to that of the competitive binding assay. Briefly, 200-μL reaction mixtures containing CCRF-CEM (0.5 × 10^6^ cells) and ^67^Ga-FRM001 with varying FRM001 concentrations were incubated at 37 °C for one hour in the presence or absence of AMD3100 (0.1 mM). Following the incubation, the cells were washed three times with the assay buffer, and radioactivity counts of the cells were determined with a gamma counter.

### Internalization assay

The internalization assay was performed in 1.5-mL centrifuge tubes using RPMI-1640 containing 5% BSA as the binding buffer. Briefly, 200 μL of reaction mixtures containing CCRF-CEM (0.5 × 10^6^ cells) and ^67^Ga-FRM001 (0.5 pmol of FRM001) were incubated at 37 °C for one hour in the presence or absence of AMD3100 (0.1 mM). After the incubation, the samples were centrifuged at 291 × g for 5 min and the supernatant was removed. To quantify the amounts of internalized radioactivity, the cells were washed twice with an acid wash buffer (0.2 M of glycine, 0.15 M of NaCl; pH: 3.0) and the binding buffer, respectively. To quantify the amounts of total cellular radioactivity, the cells were washed twice with the binding buffer. The radioactivity counts of the cells were determined with a gamma counter. Since the total numbers of cells per tube were varied by the cell washing step, the data were corrected by the number of cells.

### Preparation of tumor xenograft model

All animal experiments were conducted in accordance with the Guidelines for Proper Conduct of Animal Experiments issued by the Science Council of Japan (June 1, 2006), and all experimental protocols were approved by the Institutional Animal Care and Use Committee of FUJIFILM RI Pharma Co., Ltd. (Permit No. Rin-170802 and Rin-180101). The acute lymphoblastic leukemia CCRF-CEM model was prepared using a published procedure^36^. Briefly, 5 × 10^6^ cells mixed with Matrigel (1:1; Corning, Corning, NY) were implanted subcutaneously into the rear flank of female 10-week-old SCID mice (C.B-17/Icr-*scid*/*scid*; CLEA Japan, Tokyo). After an average of three weeks, the animals were used in biodistribution and PET imaging studies. Tumor volumes ranged from 709–2,257 mm^3^, while the tumor mass-to-body weight ratio was 3.1–9.4%.

### Biodistribution study

A biodistribution study was conducted by intravenously administering ^67^Ga-FRM001 (0.6 MBq/0.2 nmol FRM001/mouse) in the presence or absence of AMD3100 (0.06 µmol/mouse) into CCRF-CEM tumor-bearing mice. Mice were sacrificed at four hours after the injection. Organs of interest were removed, weighed, and the radioactivity counts were determined using a gamma counter. The biodistribution of each sample was expressed as a percentage of the injected dose per gram of wet tissue weight (%ID/g).

### PET imaging study

A PET imaging study was conducted by intravenously administering ^68^Ga-FRM001 (2–4 MBq/0.2 nmol FRM001/mouse) in the presence or absence of AMD3100 (0.06 µmol/mouse) into CCRF-CEM tumor-bearing mice. The static PET images were acquired for 15 min at one hour post-injection, using an Inveon PET scanner. After the reconstruction, the images were displayed by using maximum-intensity projection. The mice were anesthetized with isoflurane during the imaging.

### Statistical analysis

Biodistribution data and *in vitro* binding data were statistically analyzed by using Welch’s t-test (two-tailed test). The level of statistical significance was set at p < 0.05. The statistical analysis was performed by using EXSUS version 8.1 (CAC Croit, Tokyo).

## Results and Discussion

### Design and synthesis of FRM001

FRM001 was designed based on the tentative binding pose of LY2510924 in the CXCR4 ligand-binding cavity^32^. FRM001 was synthesized by conjugating maleimido-mono-amide-DOTA to the C-terminus of LY2510924 through a cysteine linker, followed by the preparative RP-HPLC purification (Fig. 1). FRM001 was obtained with a purity of 98.48% as confirmed by the analytical HPLC and characterized by the MALDI TOF-MS (see Supplementary Fig. S1).

### Synthesis of Ga/Lu/Y-FRM001

Ga-FRM001, Lu-FRM001, and Y-FRM001 were synthesized by incubating 20 equivalents of GaCl_3_, LuCl_3,_ and YCl_3_ over FRM001 for 30 min at 45 °C in sodium acetate buffer (pH: 5). After RP-HPLC, Ga-FRM001, Lu-FRM001, and Y-FRM001 were obtained with purities of 94.23%, 96.24%, and 96.80% as confirmed by the analytical HPLC. All products were characterized by ESI-MS (see Supplementary Figs S2–S4).

### Preparation of ^67/68^Ga-FRM001

^67^Ga-FRM001 was prepared by incubating FRM001 with ^67^GaCl_3_ for 10 min at 95 °C in a sodium acetate buffer (pH: 5), followed by the purification through a Sep-Pak C18 column. The radiochemical yield was assessed to be ≥95% by radio-TLC. The radiochemical purity was ≥96% by radio-TLC and ≥97% by radio-HPLC (see Supplementary Fig. S5). The specific activity was 3 MBq/nmol. ^68^Ga-FRM001 was prepared by incubating FRM001 with ^68^GaCl_3_ for 10 min at 95 °C in sodium acetate buffer (pH: 5) without post-labeling purification to shorten the preparation time. The radiochemical purity was assessed to be ≥98% by radio-TLC and ≥95% by radio-HPLC (see Supplementary Fig. S6). The specific activity was 19–20 MBq/nmol.

### Lipophilicity (logP_ow_)

The logP_ow_ value of ^67^Ga-FRM001 in a mixture of PBS (pH: 7.4) and 1-octanol was −3.23 ± 0.05 (the mean value ± standard deviation, triplicate).

### CXCR4 binding affinity

Since the purpose of this study was to assess *in vivo* accumulation to CXCR4-expressing cells, the apparent binding affinities were determined at 37 °C. To compare the affinity for CXCR4 under the present study, the binding affinities of the reference compounds, LY2510924, FC131, BKT140, AMD3465, and AMD3100 were also determined under similar conditions. The half-maximal inhibitory concentration (IC_50_) values of FRM001, its Ga-, Lu-, and Y-complexes, and other CXCR4 antagonists were determined with CCRF-CEM cells expressing endogenous CXCR4^34^. The displacement of ^125^I-SDF-1α bound to the CXCR4 with the compounds was also evaluated in the presence of 0.25 nM of SDF-1α. Table 1 summarizes the IC_50_ values of the compounds. The competitive binding curves are presented in Supplementary Fig. S7. FRM001 showed improved CXCR4 affinity as compared with FC131^37^, AMD3100^38,39^, AMD3465^40^, and BKT140^41,42^, which are reported to be potent CXCR4 antagonists.

**Table 1:** IC_50_ values of CXCR4 antagonists for binding of ^125^I-SDF-1α to CXCR4-expressing CCRF-CEM cells.

CXCR4 antagonist	IC_50_ (nM)
FRM001	1.78 ± 0.15
Ga-FRM001	2.26 ± 0.51
Lu-FRM001	2.15 ± 0.14
Y-FRM001	0.89 ± 0.04
LY2510924	1.37 ± 0.10
FC131	13.07 ± 1.10
BKT140	15.43 ± 1.60
AMD3465	12.83 ± 1.52
AMD3100	21.81 ± 6.58

The data are shown as the mean values ± standard deviations (n = 3).

The similar IC_50_ values between FRM001 and LY2510924 indicated that the DOTA conjugation through the C-terminus of LY2510924 via a cysteine linker preserved the inherent affinity for CXCR4. The binding affinity of FRM001 also remained unchanged after complexation with a metal ion (Ga^3+^, Lu^3+^, or Y^3+^). In contrast, the complexation of different metal ions with pentixafor or pentixather led to significant changes in CXCR4 affinities^30,31^. These results suggested that the 1,4,7,10-tetraazacyclododecane-1,4,7-triacetic acid (DO3A) moiety in FRM001 would be well-separated from the CXCR4 binding motif of the molecule via a cysteine linker, which would account for the tolerance of FRM001 to the complexation reaction with the three metal ions. Since DO3A forms kinetically inert complexes with ^67/68^Ga, ^177^Lu, ^90^Y, ^111^In, and ^225^Ac^33,43–45^, FRM001 would constitute a useful scaffold upon which to develop a variety of theranostic pairs combined with the metallic radionuclides.

### CXCR4-specific binding and internalization

The CXCR4 specificity of ^67^Ga-FRM001 was determined with CCRF-CEM cells in the presence of a CXCR4 antagonist, AMD3100. The internalization activity was also measured by washing the cells with an acid buffer to remove the receptor-bound ^67^Ga-FRM001^46^.

**Figure 2:**
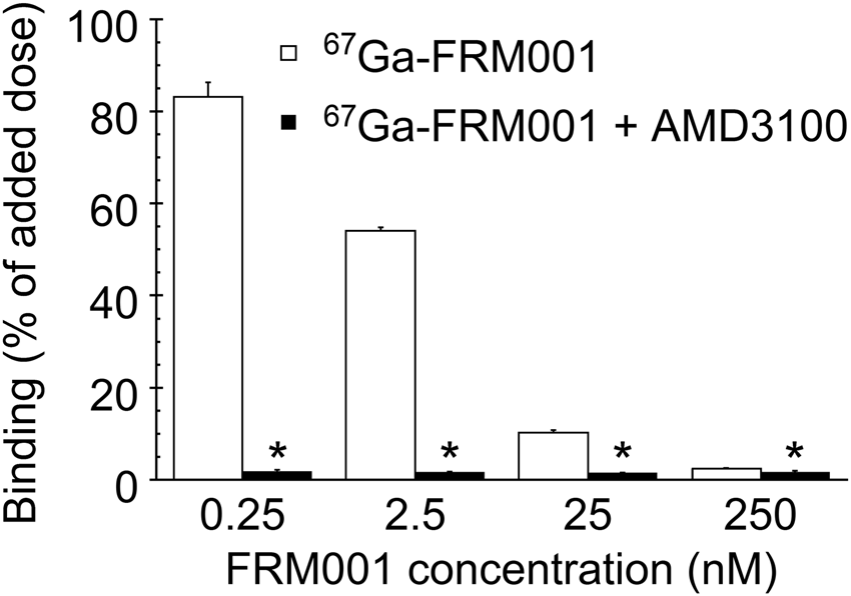
*In vitro* binding specificity of ^67^Ga-FRM001 to CCRF-CEM cells. CCRF-CEM cells (0.5 × 10^6^ cells) were incubated with ^67^Ga-FRM001 at 37 °C for one hour in the absence (white bars) and presence (black bars) of AMD3100 (0.1 mM, CXCR4 antagonist). The data are shown as the mean values ± standard deviations (triplicate). ^*^A significant difference (p < 0.05) was determined by performing Welch’s t-test.

**Figure 3:**
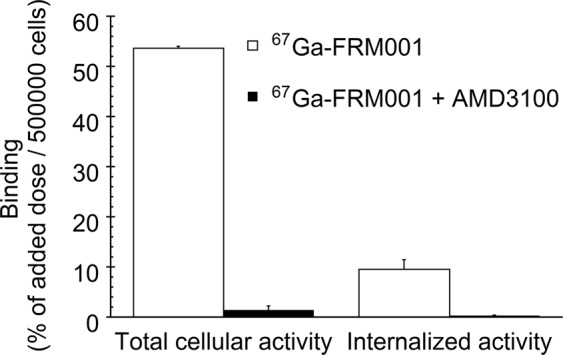
Internalization of ^67^Ga-FRM001 to CCRF-CEM cells. CCRF-CEM cells (0.5 × 10^6^ cells) incubated with ^67^Ga-FRM001 (2.5 nM of FRM001) at 37 °C for one hour in the absence (white bars) and presence (black bars) of AMD3100 (0.1 mM, CXCR4 antagonist) were washed with the binding buffer (RPMI-1640 containing 5%BSA; total cellular activity) or the acid wash buffer (0.2 M of glycine, 0.15 M of NaCl; pH: 3.0; internalized activity). The data are shown as the mean values ± standard deviations (triplicate).

^67^Ga-FRM001 was bound to CCRF-CEM cells in a dose-dependent manner and exhibited high specificity for CXCR4 (Fig. 2 and Supplementary Fig. S9). The majority of ^67^Ga-FRM001 bound to cells via CXCR4 was not internalized and remained at the cell membrane (Fig. 3). The complete inhibition of internalized ^67^Ga-FRM001 by AMD3100 (Fig. 3) also suggested that the internalization of ^67^Ga-FRM001 would only proceed after binding to CXCR4 on the cells. Similar results were observed with another antagonistic CXCR4 probe^47^. LY2510924, a CXCR4 antagonist, does not downregulate cell-surface CXCR4^48^ and inhibits SDF-1–/CXCR4-mediated intracellular signaling^32^. Thus, ^67^Ga-FRM001 would also possess an antagonistic profile and inhibit SDF-1–/CXCR4-mediated intracellular signaling similar to LY2510924.

### Stability of ^67^Ga-FRM001

**Figure 4:**
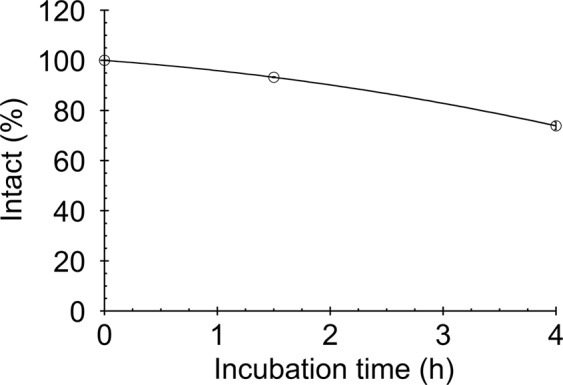
*In vitro* stability of ^67^Ga-FRM001 in serum. ^67^Ga-FRM001 incubated in human serum at 37 °C was analyzed with radio-HPLC. The data are shown as the mean values ± standard deviations (triplicate).

**Figure 5:**
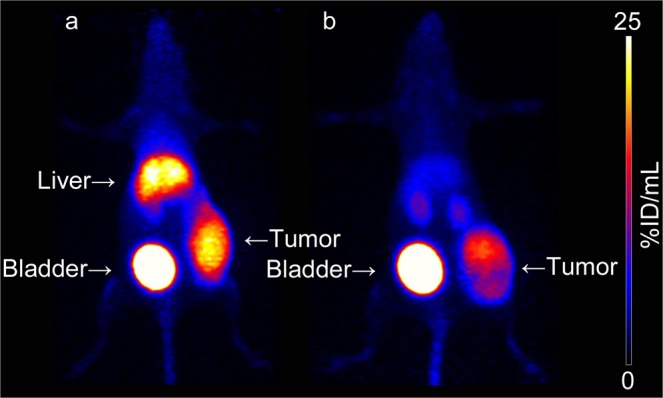
PET images of CCRF-CEM tumor-bearing mice dosed with ^68^Ga-FRM001 (2 MBq/0.2 nmol FRM001/mouse, intravenous injection). The PET images were acquired at one hour after the injection in the absence (**a**) and presence (**b**) of AMD3100 (0.06 µmol/mouse, CXCR4 antagonist). The images are shown as maximum-intensity projections.

The stability of ^67^Ga-FRM001 was determined by incubating the radioconjugate in human serum at 37 °C. More than 90% of ^67^Ga-FRM001 remained intact after 90 min incubation in human serum (Fig. 4). After four hours of incubation, the intact fraction decreased to 74%, and a degradation product was detected at a retention time slightly earlier than that of the intact ^67^Ga-FRM001 (see Supplementary Fig. S8). While further stability enhancement is preferable, FRM001 does possess serum stability sufficient for *in vivo* studies considering the rapid elimination rates of ^67^Ga-FRM001 from the blood (Table 2) and the PET imaging studies with a short half-life radionuclide ^68^Ga (Fig. 5).

**Table 2:** Biodistribution of ^67^Ga-FRM001 (0.6 MBq/0.2 nmol FRM001/mouse, intravenous injection) in CCRF-CEM tumor-bearing mice at four hours post-injection.

Organ	^67^Ga-FRM001	^67^Ga-FRM001 + AMD3100^a^
blood	0.205 ± 0.033	0.175 ± 0.009
heart	0.385 ± 0.063	0.164 ± 0.007^*b*^
lungs	2.008 ± 0.301	0.412 ± 0.034^*b*^
thymus	1.574 ± 0.587	0.228 ± 0.102^*b*^
liver	16.110 ± 2.661	4.008 ± 0.169^*b*^
spleen	3.766 ± 0.195	0.602 ± 0.029^*b*^
pancreas	0.198 ± 0.016	0.111 ± 0.005^*b*^
stomach	0.381 ± 0.417	0.098 ± 0.032
small intestine	0.355 ± 0.036	0.171 ± 0.013^*b*^
large intestine	0.852 ± 0.318	0.671 ± 0.060
fat	0.291 ± 0.137	0.096 ± 0.049^*b*^
ovary	0.808 ± 0.247	0.239 ± 0.101^*b*^
uterus	0.944 ± 0.338	0.386 ± 0.043^*b*^
muscle	0.107 ± 0.018	0.063 ± 0.007^*b*^
bone	0.682 ± 0.099	0.174 ± 0.019^*b*^
skin	0.404 ± 0.076	0.185 ± 0.023^*b*^
brain	0.015 ± 0.003	0.013 ± 0.002
adrenals	1.022 ± 0.192	0.251 ± 0.033^*b*^
kidneys	4.846 ± 0.501	6.131 ± 0.207^*b*^
tumor	12.022 ± 1.993	8.845 ± 0.873^*b*^

The data are shown as the mean percentages of the injected dose per gram of wet tissue weight (%ID/g) ± standard deviations (for each group, n = 5 mice). ^a^AMD3100 was co-injected (0.06 µmol/mouse). ^b^A significant difference (p < 0.05) was determined by performing Welch’s t-test.

### Biodistribution of ^67^Ga-FRM001

The biodistribution of ^67^Ga-FRM001 was assessed in CCRF-CEM tumor-bearing mice. Each animal was given ^67^Ga-FRM001 (0.6 MBq/0.2 nmol FRM001/mouse, intravenous injection) and sacrificed at four hours post-injection. ^67^Ga-FRM001 was also administered to another group of animals along with AMD3100 (0.06 µmol/mouse). These results are summarized in Table 2.

^67^Ga-FRM001 showed a high rate of accumulation in the tumor and rapid elimination rate from the blood. As a result, ^67^Ga-FRM001 exhibited a tumor-to-blood ratio of 59 at four hours post-injection. As shown in Fig. 3, ^67^Ga-FRM001 possesses an antagonist profile with a high binding affinity to the target molecule. Thus, ^67^Ga-FRM001 constitutes another example that supports the paradigm shift from agonists to antagonists as radioligands for *in vivo* targeting with the peptide-based probe^31,49,50^. The accumulation in the spleen, thymus, and bone would be attributable to physiological expression of CXCR4 in these tissues as reported previously^51^, which was supported by the significant reduction in the radioactivity levels of these tissues following AMD3100 co-injection (Table 2). ^67^Ga-FRM001 also displayed a high hepatic accumulation similar to those levels of other CXCR4-targeting probes such as ^68^Ga-NOTA-NFB^9,52^, ^64^Cu-AMD3100^10,53^, and ^177^Lu-pentixather^14,31^. The AMD3100 co-injection significantly reduced the hepatic radioactivity levels (Table 2) similar to in the cases of other CXCR4-targeting probes. Since CXCR4 is constitutively expressed in the sinusoidal endothelial cells of the murine liver^54^, the CXCR4-mediated accumulation would be partially involved in the hepatic accumulation. However, the CXCR4 expression levels in the liver are lower than those of the spleen, thymus, and thymus^51^. The accumulation of ^67^Ga-FRM001 in the tumor was much less inhibited by the AMD3100 co-injection when compared with that in the liver (Table 2). Considering significantly higher binding affinity of Ga-FRM001 to CXCR4 than to AMD3100, the hepatic accumulation of ^67^Ga-FRM001 would be attributable to another yet-unknown mechanism, as also reported in other CXCR4-targeting probes^7,31,55^. Recently, the involvement of organic cation transporters has been discussed as a hepatic accumulation mechanism^56^. The blockage of hepatic accumulation of ^67^Ga-FRM001 by AMD3100 co-injection would facilitate the renal excretion of free ^67^Ga-FRM001 in plasma, which was reflected in a slight but significant increase in the renal radioactivity levels (Table 2). Similar results were observed in radiolabeled CXCR4 probes^31,57^ and a ^99m^Tc-labeled bivalent RGD probe^58^.

### PET imaging study

Figure 5 shows representative PET images in CCRF-CEM tumor-bearing mice after the administration of ^68^Ga-FRM001 (2–4 MBq/0.2 nmol FRM001/mouse, intravenous injection) in the presence or absence of AMD3100 (0.06 µmol/mouse). PET images were acquired at one hour post-injection.

While ^68^Ga-FRM001 visualized the CCRF-CEM tumor, high radioactivity levels were observed in the liver (Fig. 5a). The AMD3100 co-injection significantly and preferentially reduced the hepatic accumulation. As a result, the AMD3100 co-injection significantly improved the tumor-targeting performance of ^68^Ga-FRM001 (Fig. 5b).

## Conclusions

FRM001 was developed as a new scaffold for delivering radiation to CXCR4 for imaging and endoradiotherapy. ^67^Ga-FRM001 showed the specific binding of CXCR4-expressing cells with a binding affinity similar to that of the parental LY2510924. The binding affinity of FRM001 was well-preserved after complexation with trivalent metal ions such as Ga^3+^, Y^3+^, and Lu^3+^. The high accumulation of ^67/68^Ga-FRM001 in the tumor was also confirmed in a mouse model. Although further studies of the hepatic accumulation are needed, the present findings suggest that FRM001 would be useful in developing CXCR4-targeting radiolabeled probes for molecular imaging and radionuclide therapy. The present findings also suggest that the C-terminal modification of LY2510924 may also provide CXCR4-targeting probes for other imaging modalities (e.g., magnetic resonance imaging, fluorescence imaging) and CXCR4-targeting therapeutics with cytotoxic drugs for tumor treatment.

## Supplementary information

Supplementary information

## Data Availability

The datasets generated during and/or analyzed during the current study are available from the corresponding author on reasonable request.
