# Supplementary material for: C-terminal–modified LY2510924: a versatile scaffold for targeting C-X-C chemokine receptor type 4
Source: Sci Rep. 2019 Oct 25;9:15284. doi: 10.1038/s41598-019-51754-0 (PMC6814797; doi:10.1038/s41598-019-51754-0)
Supplement: Supplementary file 1 — Supplementary information [file 41598_2019_51754_MOESM1_ESM.pdf]

Supplementary information

C-terminal–modified LY2510924: a versatile scaffold for targeting C-X-C chemokine receptor type 4

Kentaro Suzuki[*,1,2], Takashi Ui[3], Akio Nagano[1], Akihiro Hino[1] & Yasushi Arano[2]

[1] RI Research Department, Research Division, FUJIFILM Toyama Chemical Co., Ltd., 453-1, Shimo-Okura, Matsuo-Machi, Sammu-City, Chiba 289-1592, Japan

[2] Department of Molecular Imaging and Radiotherapy, Graduate School of Pharmaceutical Sciences, Chiba University, 1-8-1, Inohana, Chuo-ku, Chiba 260-8675, Japan

[3] Research Department, FUJIFILM RI Pharma Co., Ltd., 453-1, Shimo-Okura, Matsuo-Machi, Sammu-City, Chiba 289-1592, Japan

[*] Corresponding Author

E-mail: kentaro.a.suzuki@fujifilm.com; Phone: +81-479-86-4722; Fax: +81-479-86-3522

Table of contents

Figure S1. ·· S3

Figure S2. ·· S4

Figure S3. ·· S5

Figure S4. ·· S6

Figure S5. ·· S7

Figure S6. ·· S8

Figure S7. ·· S9

Figure S8. ··S10

Figure S9. ··S11

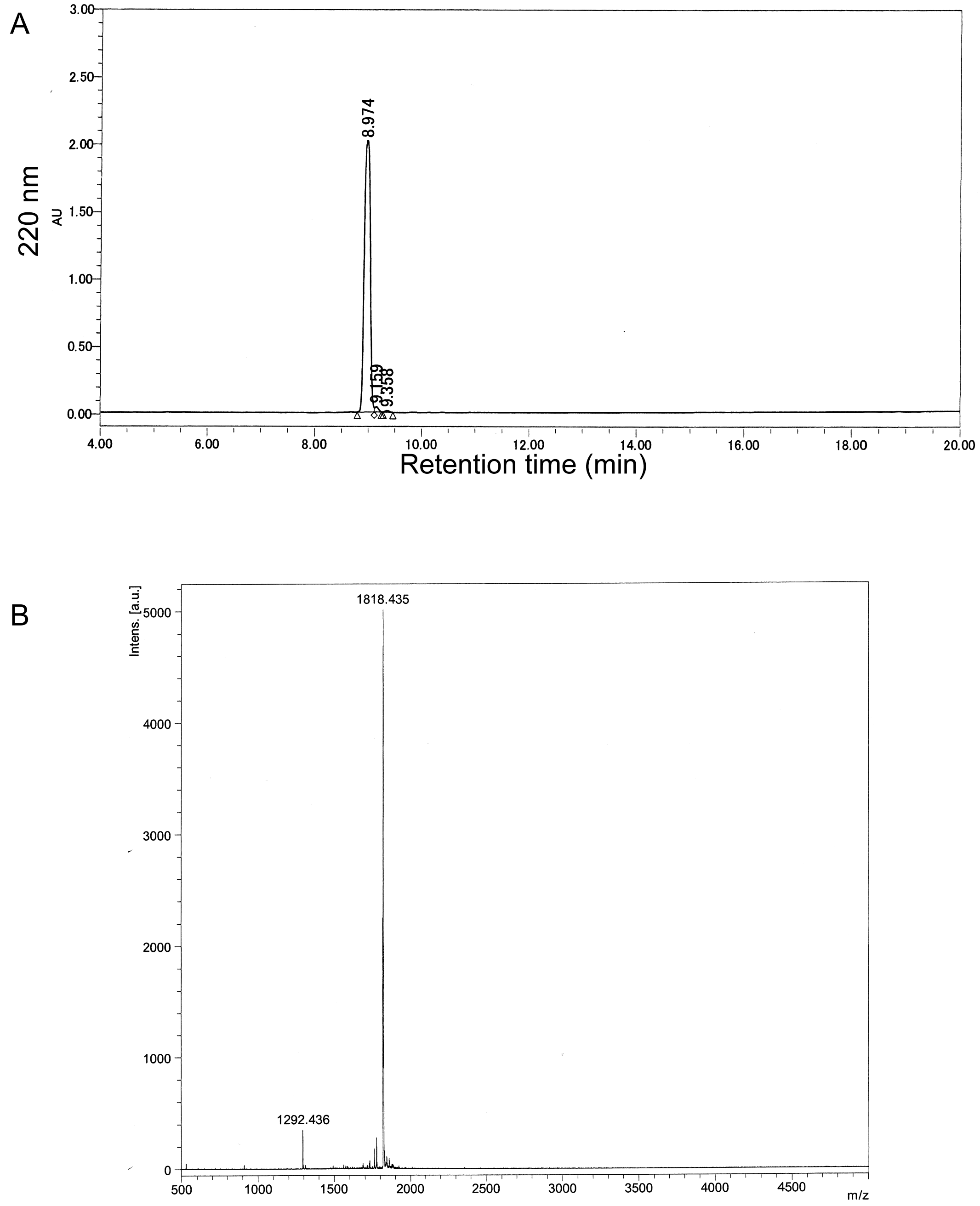

Figure S1. (A) HPLC profile of FRM001. The peak at a retention time of 9.0 min represents FRM001. The analysis was performed with a SunFire C18 column (5 μm, 4.6 × 150 mm; Waters) at a flow rate of 1 mL/min under the following linear gradient of 10%–60% acetonitrile (0.1%TFA) in water (0.1%TFA) over 20 min. (B) Mass spectrum of FRM001. MALDI TOF-MS: calculated for ($C_{87}H_{127}N_{21}O_{20}S$): 1,817.93; found: *m/z* = 1,818.435 $[M+H]^+$.

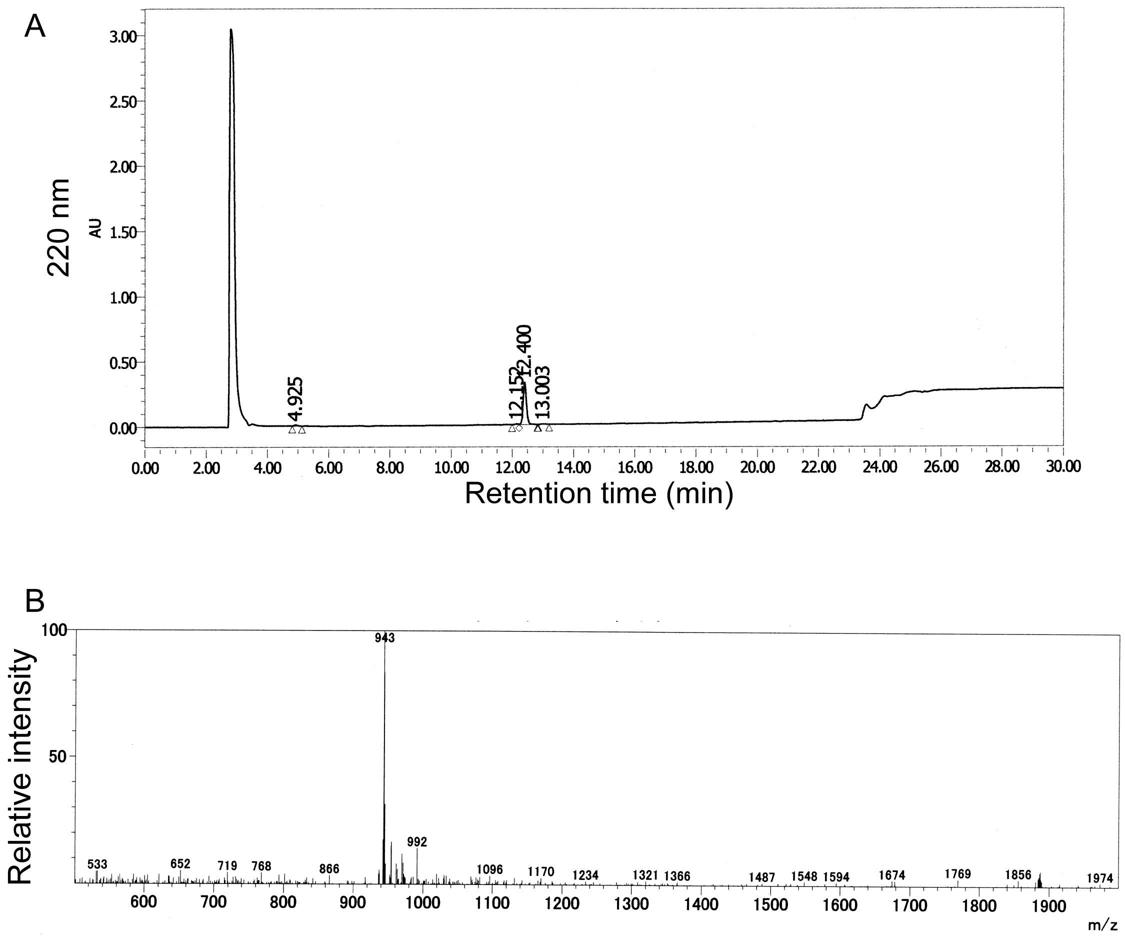

Figure S2. (A) HPLC profile of Ga-FRM001. The peak at a retention time of 12.4 min represents Ga-FRM001. The peak at a retention time of about 3 min represents dimethyl sulfoxide as a solvent. The analysis was performed by using the same method as that employed in radio-HPLC. (B) Mass spectrum of Ga-FRM001. ESI-MS: calculated for ($C_{87}H_{125}GaN_{21}O_{20}S$): 1,884.84; found: *m/z* = 943 $[M+2H]^{2+}$.

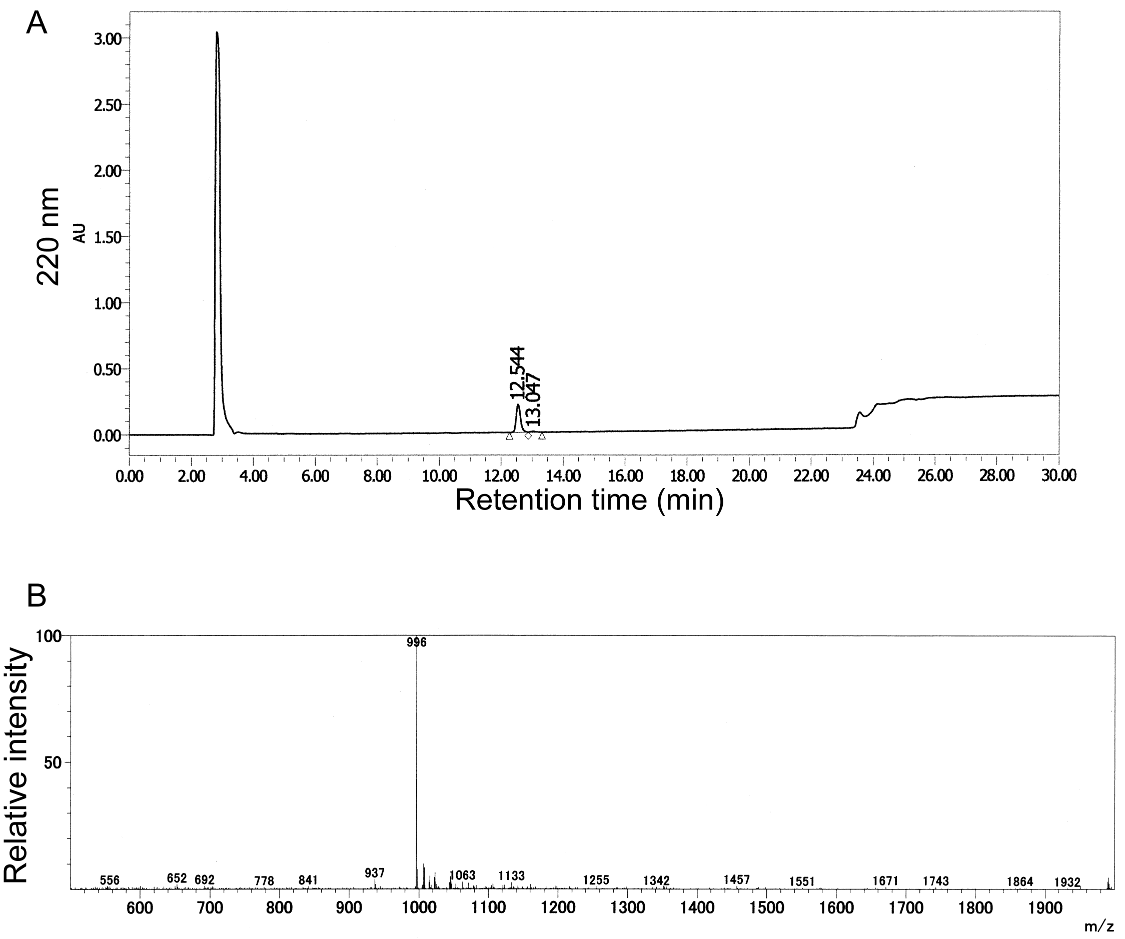

Figure S3. (A) HPLC profile of Lu-FRM001. The peak at a retention time of 12.5 min represents Lu-FRM001. The peak at a retention time of about 3 min represents dimethyl sulfoxide as a solvent. The analysis was performed by using the same method as that used in radio-HPLC. (B) Mass spectrum of Lu-FRM001. ESI-MS: calculated for ($C_{87}H_{124}LuN_{21}O_{20}S$): 1,989.85; found: *m/z* = 996 $[M+2H]^{2+}$.

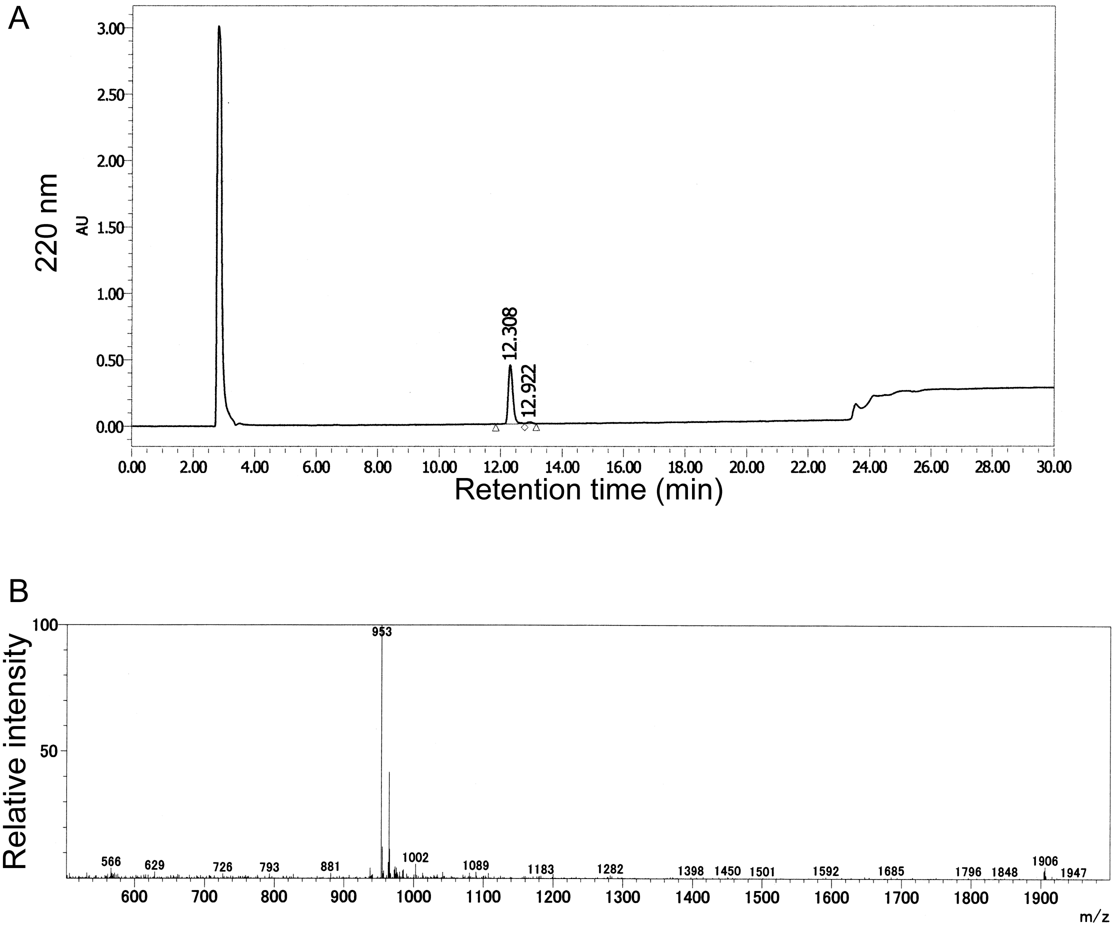

Figure S4. (A) HPLC profile of Y-FRM001. The peak at a retention time of 12.3 min represents Y-FRM001. The peak at a retention time of about 3 min represents dimethyl sulfoxide as a solvent. The analysis was performed by using the same method as used in radio-HPLC. (B) Mass spectrum of Y-FRM001. ESI-MS: calculated for ($C_{87}H_{124}N_{21}O_{20}SY$): 1,903.81; found: *m/z* = 953 $[M+2H]^{2+}$.

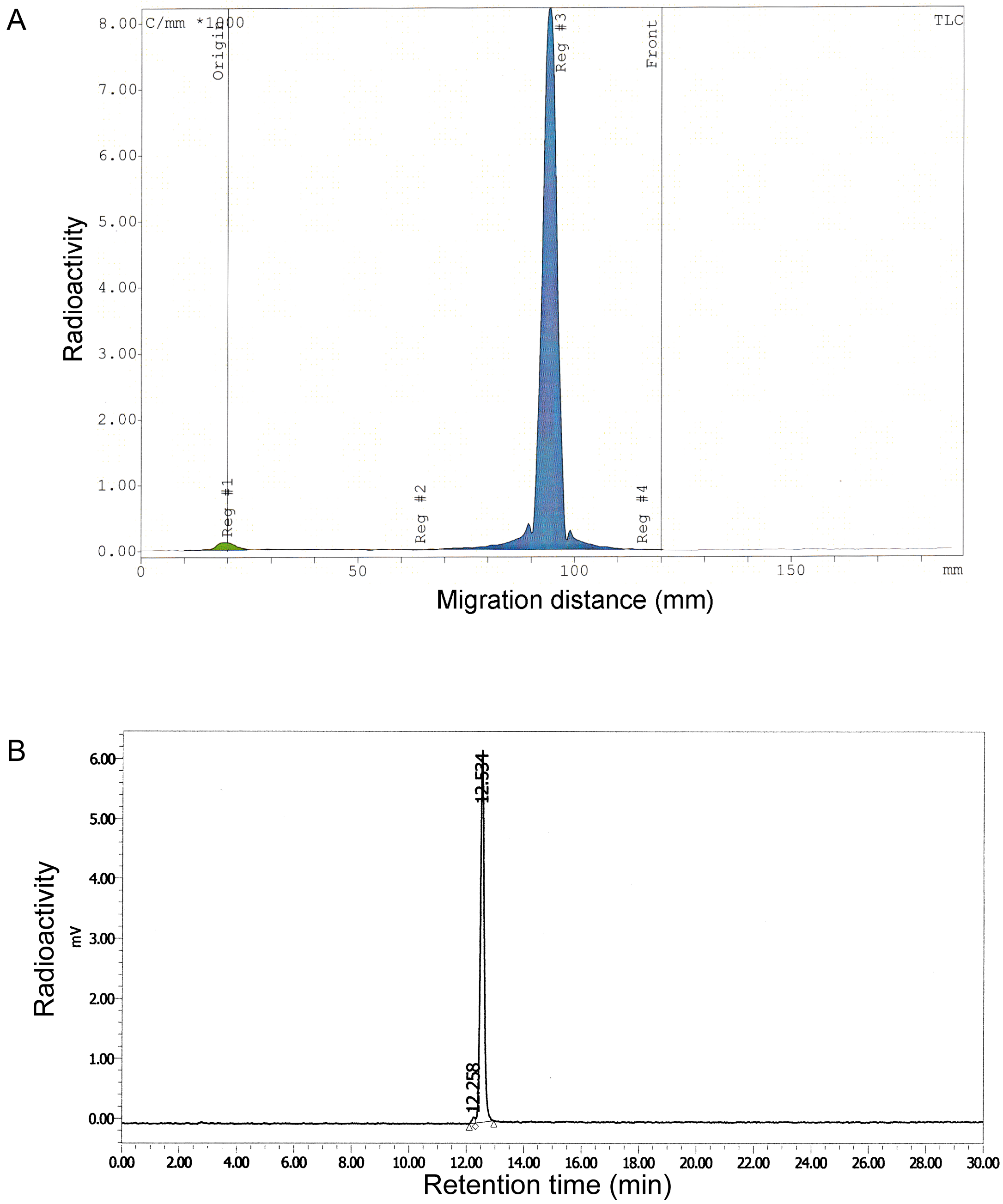

Figure S5. (A) Representative radio-TLC profile of ^{67}Ga-FRM001. The peak at an R_f value of 0.7–0.9 represents ^{67}Ga-FRM001. The peak detected at the origin represents free ^{67}Ga. (B) Representative radio-HPLC profile of ^{67}Ga-FRM001. The peak at a retention time of 12.5 min represents ^{67}Ga-FRM001.

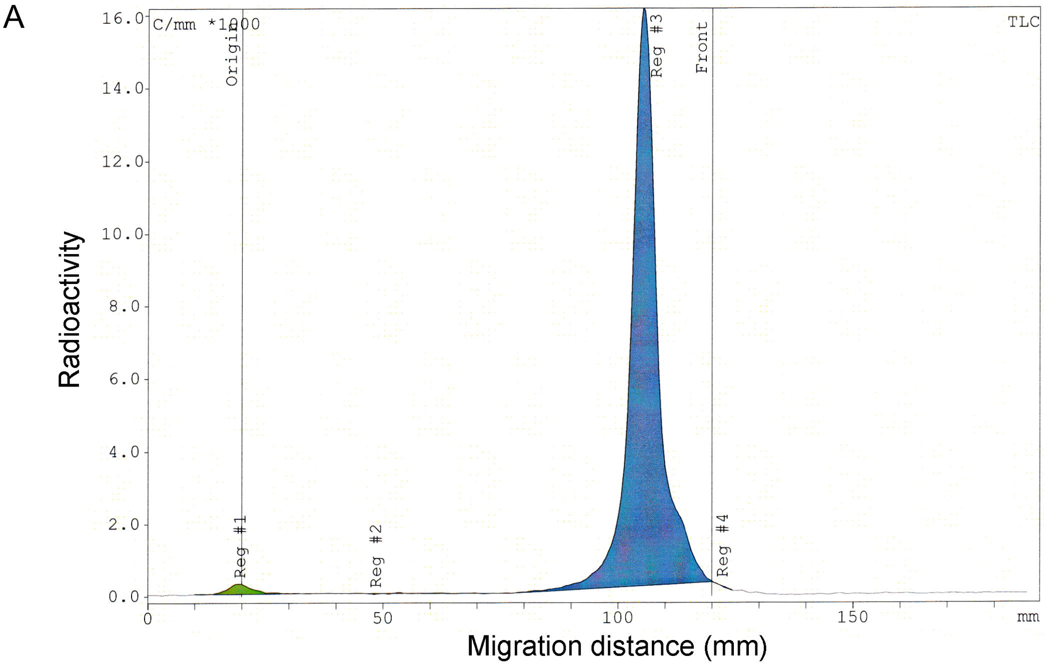

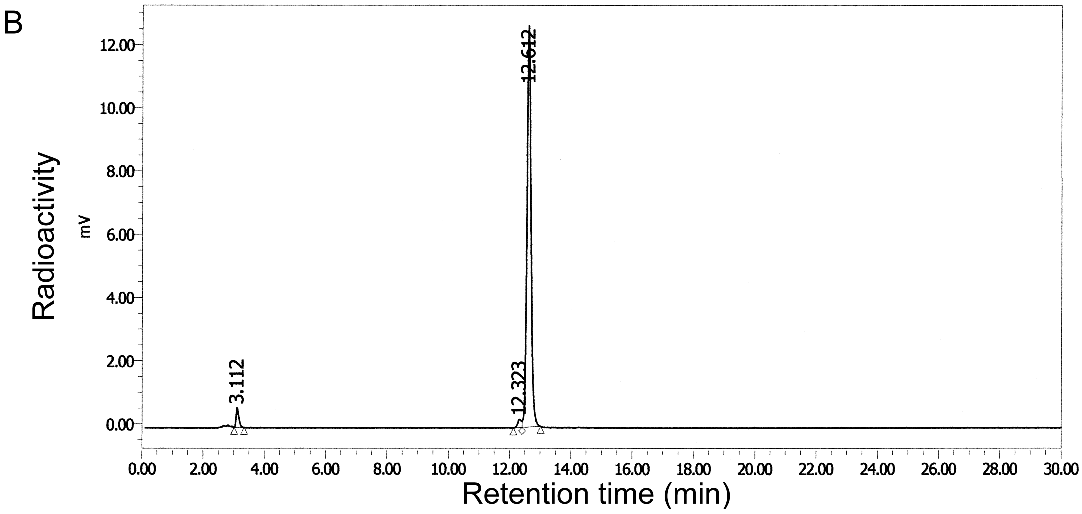

Figure S6. (A) Representative radio-TLC profile of ^{68}Ga-FRM001. The peak at an R_f value of 0.7–0.9 represents ^{68}Ga-FRM001. The peak detected at the origin represents free ^{68}Ga. (B) Representative radio-HPLC profile of ^{68}Ga-FRM001. The peak at a retention time of 12.6 min represents ^{68}Ga-FRM001. The peak detected at a retention time of 3.1 min represents a side-product from the thermolysis or free ^{68}Ga.

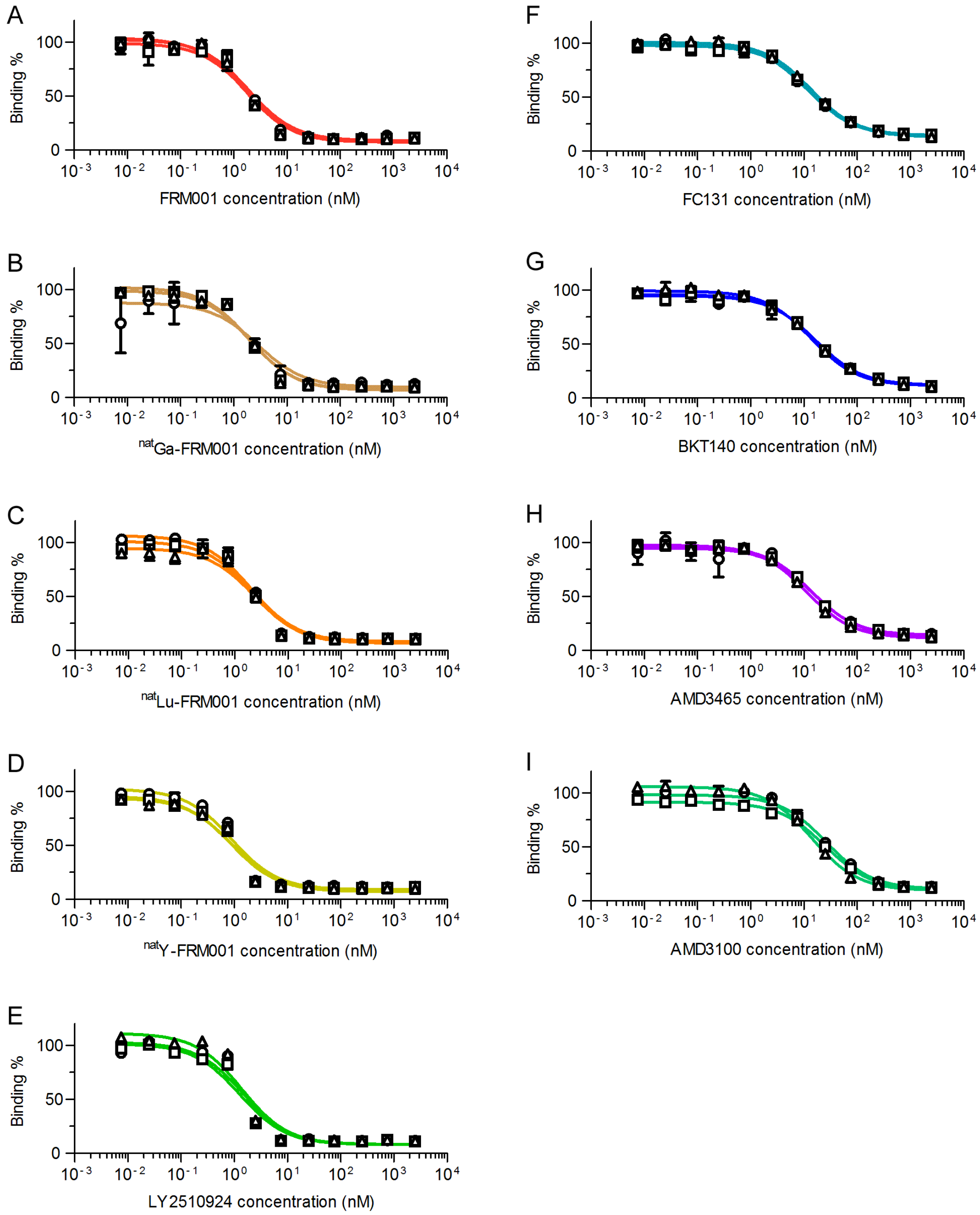

Figure S7. Inhibition of ^{125}I-SDF-1α binding to CXCR4-expressing CCRF-CEM cells by CXCR4 antagonists. The data are shown as the mean values ± standard deviations (triplicate). IC_{50} values of FRM001 (A), Ga-FRM001 (B), Lu-FRM001(C), Y-FRM001 (D), LY2510924 (E), FC131 (F), BKT140 (G), AMD3465 (H), and AMD3100 (I) were measured by three independent experiments (circle, triangle, and square).

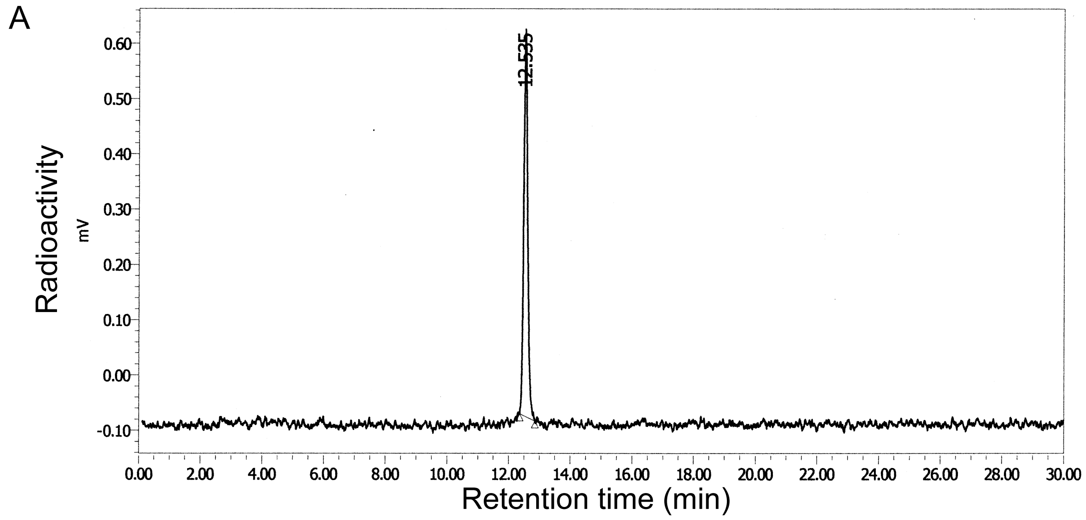

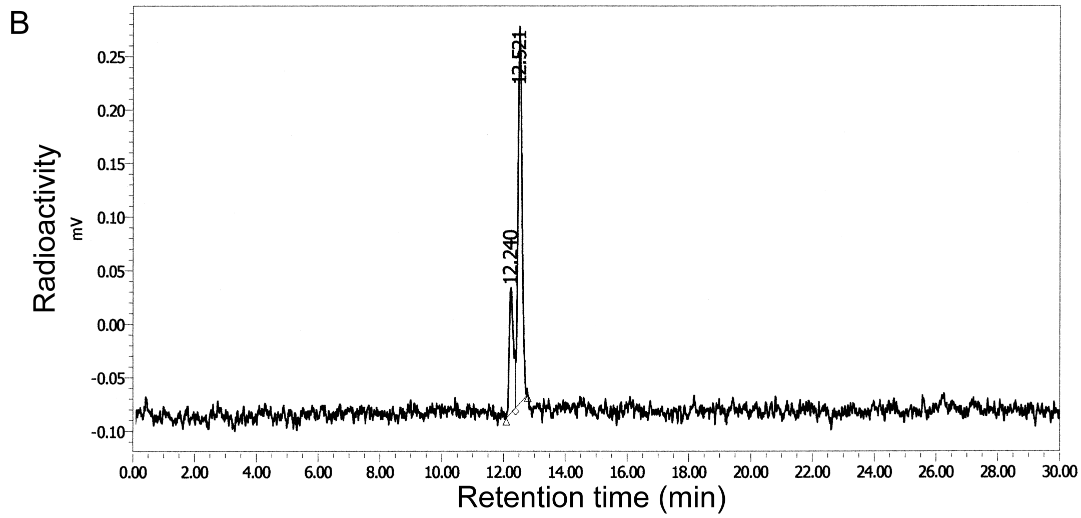

Figure S8. Representative radio-HPLC profile of ^{67}Ga-FRM001 in human serum before (A) and after four hours of incubation at 37°C (B). The peak at a retention time of 12.5 min represents ^{67}Ga-FRM001 (intact). The peak detected at a retention time of 12.2 min represents a degradation product.

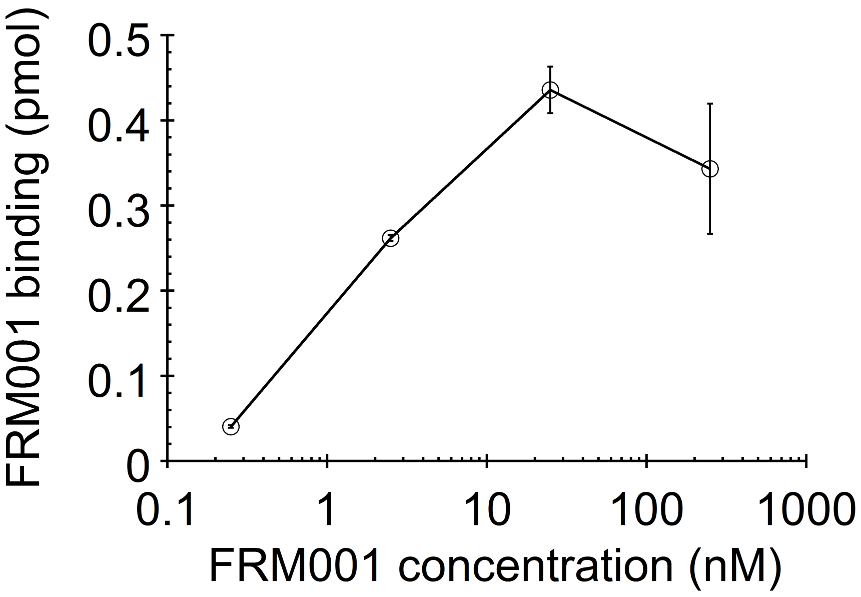

Figure S9. In vitro binding of ^{67}Ga-FRM001 to CCRF-CEM cells. Fig. S9 is the figure that re-analyzed the data of Fig. 2. The data are shown as the mean values ± standard deviations (triplicate). The y-axis was calculated as follows: [FRM001 binding (pmol)] = [FRM001 concentration (nM)] × [assay volume (0.2 mL)] × [CXCR4 specific binding of ^{67}Ga-FRM001 (% of added dose)]/100.
